# The influence of musculoskeletal pain disorders on muscle synergies—A systematic review

**DOI:** 10.1371/journal.pone.0206885

**Published:** 2018-11-05

**Authors:** Bernard X. W. Liew, Alessandro Del Vecchio, Deborah Falla

**Affiliations:** 1 Centre of Precision Rehabilitation for Spinal Pain (CPR Spine), School of Sport, Exercise and Rehabilitation Sciences, College of Life and Environmental Sciences, University of Birmingham, Edgbaston, Birmingham, United Kingdom; 2 Neuromuscular Research & Technology, Department of Bioengineering, Faculty of Engineering, Imperial College London, Kensington, London, United Kingdom; University of Illinois at Urbana-Champaign, UNITED STATES

## Abstract

**Background:**

Musculoskeletal (MSK) pain disorders represent a group of highly prevalent and often disabling conditions. Investigating the structure of motor variability in response to pain may reveal novel motor impairment mechanisms that may lead to enhanced management of motor dysfunction associated with MSK pain disorders. This review aims to systematically synthesize the evidence on the influence of MSK pain disorders on muscle synergies.

**Methods:**

Nine electronic databases were searched using Medical Subject Headings and keywords describing pain, electromyography and synergies. Relevant characteristics of included studies were extracted and assessed for generalizability and risk of bias. Due to the significant heterogeneity, a qualitative synthesis of the results was performed.

**Results:**

The search resulted in a total of 1312 hits, of which seven articles were deemed eligible. There was unclear consistency that pain reduced the number of muscle synergies. There were low consistencies of evidence that the synergy vector (W weights) and activation coefficient (C weights) differed in painful compared to asymptomatic conditions. There was a high consistency that muscle synergies were dissimilar between painful and asymptomatic conditions.

**Conclusions:**

MSK pain alters the structure of variability in muscle control, although its specific nature remains unclear. Greater consistency in muscle synergy analysis may be achieved with appropriate selection of muscles assessed and ensuring consistent achievement of motor task outcomes. Synergy analysis is a promising method to reveal novel understandings of altered motor control, which may facilitate the assessment and treatment of MSK pain disorders.

## Introduction

Musculoskeletal (MSK) pain disorders are a group of disorders associated with nociception experienced within the MSK system (muscles, ligaments, joints, and tendons) [1]. These disorders have a substantial impact on an individual’s quality of life [2]. Although the resolution of pain is the primary aim of most interventions [3–5], some conservative treatments such as exercise have the additional aim of enhancing or restoring motor function [6, 7]. An important premise behind exercise interventions is that disturbed motor control not only occurs because of pain, but also contributes to pain [8–10].

The relationship between pain and altered motor control in MSK pain disorders has been commonly investigated on individual muscles [11–13], or on a small number of muscles [9, 14, 15], using movement assessments requiring a small motor solution subspace [16, 17]. A common finding of many studies which investigated the influence of MSK pain on individual muscle control is the large variability between-individuals and between-motor tasks [10, 18]. This is perhaps unsurprising given that assessing motor control within a small motor solution subspace during pain ignores the number of dimensions of the neuromuscular system (i.e. the Degree of Freedom (DOF) Problem of Bernstein [19, 20]). In the DOF Problem, the central nervous system (CNS) has to cope with an apparent excess of muscles needed to perform a given task. One solution thought to be used by the CNS to solve the DOF Problem, is to reduce the number of dimensions into discrete sets of muscle groups, known as muscle synergies [21, 22]. Understanding the behavioural effects of MSK pain without biomechanical constraints and therefore in the framework of muscle synergies may elicit more consistent patterns of motor control adaptations, than analysis within a limited motor solution subspace.

In recent years, an increasing number of studies have quantified changes in muscle synergies during either experimentally-induced pain (e.g. intramuscular injection of hypertonic saline [23]) or in clinical MSK pain populations [24, 25]. Matrix decomposition is a common dimension reduction technique used in muscle synergy analysis [26, 27], to quantify how individual muscles function as a unit, and how different functional units are coordinated to perform a motor task. In pain-free conditions, patterning of muscle synergies can help to distribute mechanical stress within and between MSK tissues [8], and to increase the number of available motor solutions for a task. Although changes in the normal patterning of muscle synergies with pain may help in transiently reducing the mechanical stress on the injured/painful MSK tissues [28], its persistence can contribute to chronic pain by increasing the magnitude of mechanical stress imposed on previously healthy MSK tissues [28]. Alteration in normal muscle synergies can also have adverse consequence on motor performance, although this has not been investigated in the context of MSK disorders [29].

To better understand the influence of MSK pain on muscle synergies, the conduct of a systematic review is critical. This is because inconsistencies in how pain affects muscle synergies between studies could be due to variations in study methodology [30], rather than true motor control variations. We anticipate that findings from this review will have significant impact for clinical practice and for stimulating new advances in research methodologies in the area of motor control in MSK disorders. For example, analogous to research in neurological populations [31–33], it is anticipated that muscle synergy analysis can be used to provide prognostic “biomarkers” of recovery and injury risk assessment, as well as drive novel and more effective therapeutic approaches that are directly based on the physiological recruitment of motor modules by the CNS. In addition, changes in muscle synergies common to multiple pain phenotypes can be potentially used for screening of MSK disorders, where the focus is on sensitivity; whilst changes in synergies specific to a particular pain phenotype may be used for diagnostic purposes-where the focus is on specificity.

Based on anecdotal knowledge of the relatively recent focus of muscle synergy analysis in MSK pain disorders, this review included studies investigating both clinical and human experimental models of pain [23]. In this review, we defined human experimental pain models as those that transiently give rise to pain in healthy individuals under controlled laboratory conditions, via the stimulation of peripheral nociceptors [23]. Herein, we proposed five sub-aims: when compared to a healthy group/condition, does MSK pain differentially influence: 1) the number of muscle groups extracted or the percentage variance (VAF) accounted for; 2) the weighting of muscles (“W” weights) within each synergy; 3) the activation coefficients (“C” weights) within each synergy; 4) similarity of the W and C weights of synergies in the presence of pain; and 5) reconstruction quality of the measured muscle activity in both conditions using only the W weights from the asymptomatic group/condition? Reconstruction quality represents how well the original EMG signal can be reconstituted using only the extracted muscle synergies and is typically represented as a percentage ratio between the reconstructed EMG over the original EMG signal. In the present review, the term “condition” will henceforth be used to collectively indicate a group in clinical studies and a condition in experimental pain studies.

## Materials and methods

The report of this study was in accordance with the Preferred Reporting Items for Systematic Reviews and Meta-Analyses (PRISMA) guidelines, the protocol of which was prospectively registered on PROSPERO (No: CRD42018081211). The PRISMA checklist can be found in the supporting information (S1 File).

### Search strategy

The following electronic databases were searched from inception to 8^th^ December 2017: Medline (Ovid), Medline (Pubmed); Embase (Ovid), CINHAL (EbscoHost), CENTRAL (Cochrane Wiley), PSYCInfo (Ovid), SportsDiscus (EbscoHost), and AMED (EbscoHost), including a grey literature search engine (http://www.greylit.org/about). References within the included studies were manually searched and forward citation tracking of the included papers was performed using SCOPUS. Medical subject headings and keywords to the following terms of pain, electromyography, synergy, and humans were used and tailored for each search engine (S1 Table). The search string was developed in consultation with co-author D.F. who has 15 years of experience in neuromuscular physiology in MSK disorders. Citations from the search engines were exported into EndNote (version X8.1, Clarivate Analytics) referencing software.

### Inclusion and exclusion criteria

Duplicates in citations were first removed, and the titles, abstracts, and full text (if needed) were screened (B.L. and A.D.V.), with a third reviewer (D.F) available for settling any disagreements, based on the following criteria below.

#### Inclusion criteria

1. Full-text journal articles (i.e. complete introduction, methods, results and discussion sections).
2. Investigated muscle synergies in either: 1) cross-sectional studies using an experimental pain paradigm, with baseline data collected in a pain-free (healthy) condition, or 2) case-control, prospective-cohort, or randomized controlled trial studies with MSK pain disorders and asymptomatic controls.
3. Investigated MSK pain
4. Analysed muscle synergies using matrix decomposition methods (e.g. non-negative matrix factorization (NNMF) or principal components analysis (PCA)).

#### Exclusion criteria

1. Conference proceedings (unless published with sufficient depth that fulfilled point (1) of the inclusion criteria)
2. Unpublished manuscripts, non-primary journal publications such as systematic reviews, non-journal publications such as books.

### Generalizability and risk of bias assessment

Two reviewers (B.L and A.D.V) independently assessed the generalizability and risk of bias scores for each study, with a third reviewer (D.F) available for settling any disagreements. We defined generalizability as the ability of each study’s methods to answer the questions raised by this review (S2 Table). Inter-rater agreement in the assessments of generalizability and risk of bias were assessed using two measures of percentage agreement (ranging from 0% [no agreement] to 100% [perfect agreement]) [34] and Gwet’s AC1 (ranging from -1 [complete disagreement] to 1 [complete agreement]) [35, 36].

In order to assess the generalizability of each study, aspects of “Population” (P), “Intervention” (I), and “Outcomes” (O) were assessed similarly to the PICOT framework commonly used in randomized controlled trials of intervention studies. For the “P” criterion, each study must provide sufficient details of the participant characteristics or the experimental pain protocol. In the “I” criterion, details about the motor tasks must be provided in sufficient depth to allow study replication. Lastly, in the “O” criterion, sufficient details on the report of instrumentation of the participants must be presented.

A custom risk of bias assessment tool, rather than a generic quality appraisal tool [34, 37], was created to address specific risk of bias of studies included in this review (S3 Table). “Selection” bias can arise when pain was not the only differentiating factor between groups at baseline prior to testing, and “performance” bias can arise when differential factors other than pain were introduced during testing. “Attrition” bias can be introduced when participants who withdrew from the study have different motor control patterns from participants who completed the study. “Reporting” bias can arise from a selective reporting only of significant results. “Detection” bias can arise from the choice of EMG signal processing methods, such as: low pass filtering frequency [30, 38], high pass filter frequency [39], number and selection of muscles [40], and the use of averaging versus concatenation in data matrix input preparation [41, 42]. For the overall risk of bias, each study was judged as a “low” risk of bias when all criteria were scored as “low”; an “unclear” risk of bias was judged when at least one criteria was scored as “unclear”; and a “high” risk of bias was judged when at least one criteria was scored as “high” [37].

### Data extraction

Information regarding the participant’s characteristics, motor task investigated, signal processing of electromyography (EMG) data, input parameters and algorithms used in matrix decomposition, and study outcomes were extracted by two reviewers (B.L., A.D.V.). A third reviewer (D.F) was available for settling any disagreements in data extraction. Five outcomes were extracted from each study. First, the number of extracted muscle synergies between conditions for a fixed VAF, or the VAF per condition for a fixed synergy number, were extracted between conditions. To aid in data synthesis, we interpreted the latter as the indication of an altered synergy number should the VAF have been fixed. For the second and third outcomes, the loading of each assessed muscle on each synergy (W weights), as well as the activation coefficients (C weights) of each synergy were extracted for each condition. For the fourth outcome, similarity measures of the W and C weights of extracted synergies in a painful condition were extracted and compared to that of the reference, asymptomatic condition. Lastly, the reconstruction quality of the measured EMG activities in both symptomatic and asymptomatic conditions were compared when using fixed W weights of synergies from the asymptomatic condition.

### Synthesis method

A meta-analysis was not performed in this review due to the large heterogeneity between studies in study design; number of muscles used to extract muscle synergies; the amount of variance needed to be accounted for as a criterion to decide the number of extracted synergies; and differences in outcome measures used (e.g. normalized dot product [24] vs Pearson cross-correlation [43]. Hence, a qualitative synthesis of results across the included studies was performed. Availability of numerical estimates of mean and standard deviation from the included studies enabled the standardized mean difference to be calculated and plotted for the outcomes of VAF and reconstruction quality. The “meta” package in R software was used to calculate the standardized mean difference (where available) for each study, each sub-task within the study, and for each pairwise comparison between pain and asymptomatic conditions [37, 44, 45].

For all five extracted outcomes, the influence of pain on the direction of effect was scored for each study: “=“ when there was no statistically significant effect of pain relative to an asymptomatic condition; “NR” when the outcome or the statistical finding was not reported; and “Δ” when there was a difference in the outcome between the symptomatic and asymptomatic condition [46]. The consistency of the association between pain and muscle synergy outcomes was assessed, and is defined as the percentage ratio of studies that agreed on the direction of association over the number of studies that reported the outcome [46]. The following criteria was used: “no” consistency when less than 33% of studies which reported the outcome agreed on the direction of association; “unclear” consistency when 34% - 60% of studies which reported the outcome agreed on the direction of association; and “high” consistency when 60% -100% of studies which reported the outcome agreed on the direction of association [46].

## Results

### Search results

**Fig 1:**
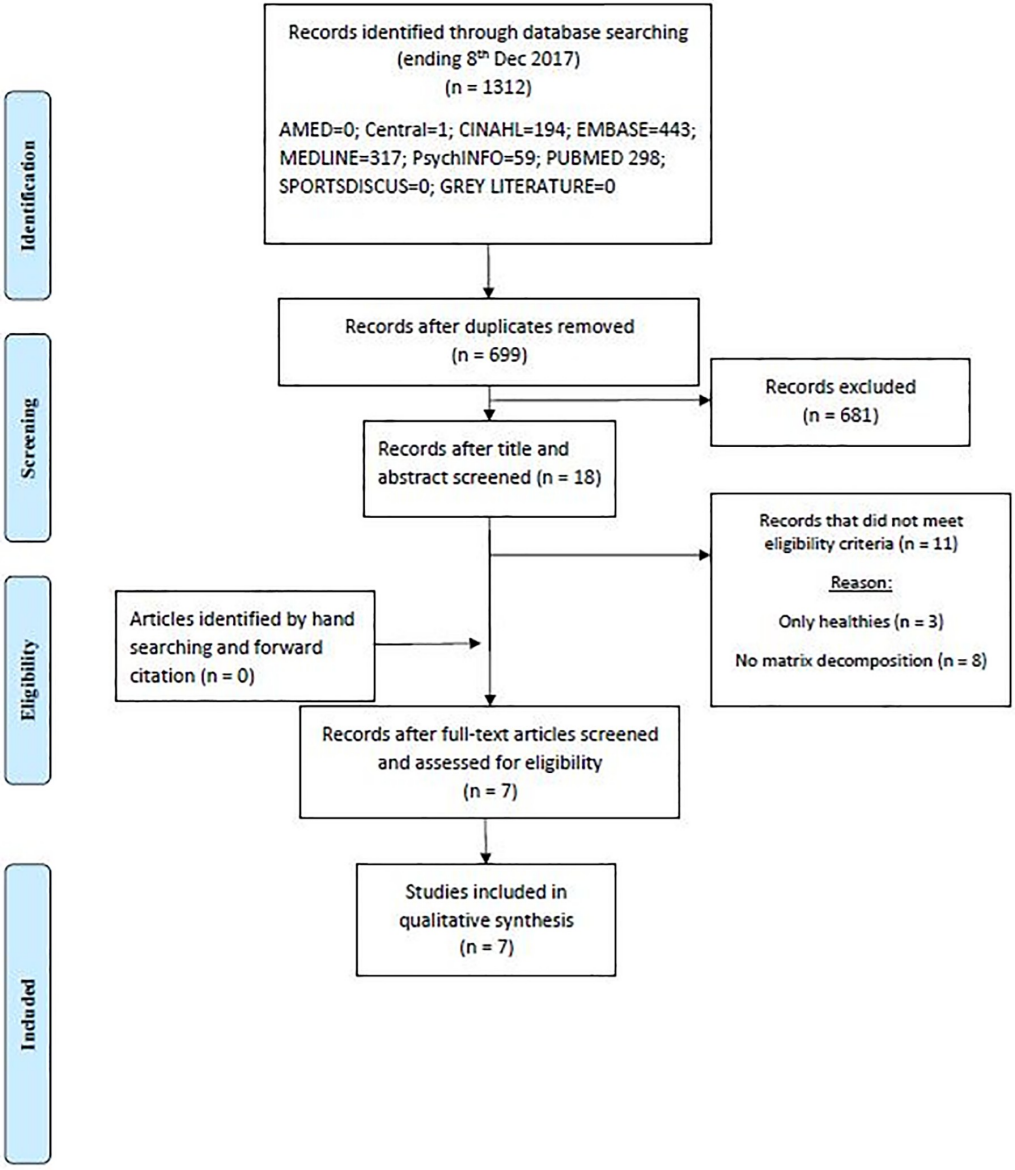
PRISMA flowchart.

An extensive search on the electronic databases yielded a total of 1312 hits (Fig 1). After duplicates were removed, and following the screening of the titles and abstracts, a total of 18 articles remained for full-text examination. Three articles that investigated only healthy participants [47–49] and eight articles that did not use matrix decomposition methods [50–57] were excluded from the review. A total of seven articles fitted all inclusion and exclusion criteria, and were included in the analysis (Fig 1).

### Study characteristics

#### Design

Three studies used a clinical case-control, cross-sectional design [25, 58, 59], three studies used a repeated measures experimental pain design [24, 43, 60], and one study used a clinical case-control, with repeated measures design [61] (Table 1). One study performed synergy analysis before and after treatment (surgery) in a clinical population, which explains the repeated measures design [61] (Table 1). Three studies investigated muscle synergies on the upper limb [25, 58, 60], one on the lower limb [59], two on the spinal region [24, 61], and one on a combined spine-lower limb region [43] (Table 1).

**Table 1:** Characteristics of included studies.

Study	Design	Pain phenotype	Demographics	Task
Diamond et al. 2016	Case-control, cross-sectional	FAI	**FAI (mean [sd])** 11 M, 4 F; 24.7 (4.9) yo; 176 (9) cm; 76 (11.8) kg **Control (mean [sd])** 8 M, 3 F; 26.6 (4.9) yo; 177 (8) cm; 72.9 (11.6) kg	Self-selected overground walking with an average speed of 1.4m/s
Heales et al. 2016	Case-control, cross-sectional	LE	**LE (mean [sd])** 4 M, 8 F; 51.6 (37 to 62) yo; ht + wt = NR; pain free grip affected arm of 81.7 (46.6) N **Control (mean [sd])** 5 M, 9 F; 51.4 (39 to 67) yo; ht + wt = NR;; grip strength of matched arm of 298.8 (76.8) N	Supported sitting, hand gripping of 20% MVC in 4 positions
Gizzi et al. 2015	Repeated measures •Base = no injection •Control = isotonic saline •Pain = hypertonic saline •Post = 10 min after Pain	Hypertonic saline injection into right Splenius Capitis	**Sample [mean (sd)]** 8 healthy; Gender = NR; 24.1 (1.9) yo; 171.6(14.7) cm; 65.6 (16) kg	Seated, multi-directional, multi-planar head tracking task to 8 targets
Wang et al. 2015	Case-control, cross-sectional & repeated measures	Low back pain; underwent single-level spinal fusion	**LBP [mean (sd)]** 10 M, 9 F; 61 (12) yo; 160(8) cm; 67 (11) kg **Control (mean [sd])** 8 M, 11 F; 60 (12) yo; 160 (7) cm; 61 (10) kg	Standing, forward reaching task with both arms
van den Hoorn et al. 2015	**Repeated measures** •Base = no injection •LBP = hypertonic saline •CalfP = hypertonic saline •PostCalfP: 4min after 100% recovery from CalfP	Hypertonic saline injection into right erector spinae at the level of the third lumbar vertebrae and right medial gastrocnemius	**Sample [mean (sd)]** 11 M, 6F; 21 (2) yo; 173(10) cm; 66 (11) kg	Fixed speed treadmill walking at 0.94m/s
Muceli et al. 2015	**Repeated measures** •Base = no injection •Control = isotonic saline •Pain = hypertonic saline: •Post = 100% pain recovery	Hypertonic saline injection into right anterior deltoid	**Sample [mean (sd)]** 8 M; 29.3 (5.3) yo; ht+wt = NR	Multidirection, horizontal reaching right arm to 12 targets spaced along a circumference
Manickaraj et al. 2017	Case-control, cross-sectional	LE	**LE (mean [sd])** 8 M, 8 F; 42 (11) yo; 174 (9) cm; 80 (16) kg; pain free grip affected arm of 126 (102) N **Control (mean [sd])** Age, sex, limb matched–Values NR; grip strength of matched arm of 246 (99) N	Supported sitting, hand gripping of 15% and 30% MVC

Abbreviations

**Outcomes: sd** = standard deviation; **mo** = months; **yo** = years old; **NA** = not applicable; **ht** = height; **wt** = weight; **PRTEE** = patient rated tennis elbow evaluation; **ODI** = Oswestry disability index; **N** = Newtons; **cm** = centimetre; **kg** = kilograms; **min** = minute; **m/s** = metre per second; **NR** = not reported; **MVC** = maximal voluntary contraction

**Clinical: FAI** = femoral acetabular impingement; **LE** = lateral epicondylalgia; **LBP** = low back pain; **M** = male; **F** = female; **CalfP** = calf pain

#### Participants

The number of participants enrolled in each study varied from eight [60] to twenty-six [59]. Five studies included both male and female participants [25, 43, 58, 59, 61], one study included only male participants [60], and one study did not report the gender of the participants [24] (Table 1). Of the four studies which investigated muscle synergies in a clinical population, three studies reported minimal pain during the motor tasks [25, 58, 59], while one study did not explicitly report pain intensity during the task performance [61]. The three studies which used an experimental pain design induced a pain intensity of more than 3/10 on a numerical rating scale (0 being no pain, and 10 being worst pain) [24, 43, 60].

#### Motor task used & matrix decomposition method

The motor tasks investigated included gait [43, 59], hand gripping [25, 58], upper limb pointing [60], forward reaching [61], and head pointing [24] (Table 1). The number of muscles used as input for matrix decomposition ranged from five [59] to 17 [43] (Table 2). Six studies used NNMF to identify muscle synergies [24, 25, 43, 58–60], with one study using Principal Components Analysis (PCA) [61] (Table 2).

**Table 2:** Electromyography assessment and synergy analysis.

Study	No. muscles assessed	No. muscles used in synergy analysis	Muscles used in synergy analysis	Filtering frequency	Amplitude normalization	Concatenating vs averaging	Method, algorithm
Diamond et al. 2016	8	5	pGMed, PI, OI, QF, SM	High-pass: 50Hz for fine-wire, 20Hz for surface electrodes Low-pass: 6Hz	Normalized to average of peak values across 3 cycles	EMG from 3 gait cycles (101 normalized points) concatenated	NNMF, Lee and Seung
Heales et al. 2016	6	6	ECRB, ECRL, EDC, FCR, FDS, FDP	Band-pass: 20 to 950Hz Low-pass: 6Hz	Normalized to average of peak values across all repetitions	EMG (200 time normalized points) for 4 positions concatenated Number of repetitions per position used: NR	NNMF, Lee and Seung
Gizzi et al. 2015	12	12	HYO, STER, SCA, SPL, UTR, LTR (bilateral)	Low-pass: 1Hz	NR	EMG (200 time normalized points) for 8 targets concatenated	NNMF, Lee and Seung
Wang et al. 2015	16	16	RA, RF, TA, ES, MF, GMAX, BF, GM (bilateral)	Band-pass: 10 to 450Hz Low-pass: 50Hz	Normalized to average RMS	EMG from 5 repetitions, unknown if concatenated vs averaging	PCA
van den Hoorn et al. 2015	19	15–17	TA, SOL, GM, GL, VM, VL, RF, BF, SM, GMAX, GMED, TFL, ES (at L3), OI, OE, IL (L3), LO (T12)	Band-pass: 20-750Hz for surface, 50-750Hz fine wire electrodes Low-pass: 9Hz	Normalized to average of peak values across the 15 cycles of the control condition	EMG from 15 cycles (each time normalized to 200 points) concatenated	NNMF, Lee and Seung
Muceli et al. 2015	12	11–12	BR, ANC, mBB, lBB, Brac, lTB, longTB, mDEL, PM, aDEL, pDEL, LD	Band-pass: 20-400Hz Low-pass: 1Hz	Not normalized	EMG (resampled to 40Hz) for 12 targets concatenated	NNMF, Lee and Seung
Manickaraj et al. 2017	6	6	ECRB,ECU, EDC, FCR, FCU, FDS	Band-pass: 10-400Hz Low-pass: 10Hz	Normalized to peak activation of same muscle using MVC	EMG (each time normalized to 500 points) for 5 trials for each of 3 conditions concatenated.	NNMF, Lee and Seung

Abbreviations

**Muscles: pGMed** = posterior gluteus medius; **PI** = piriformis; **OI** = obturator internus; **QF** = quadratus femoris; **SM** = semimembranosus; **ECRB** = extensor carpi radialis brevis; **ECRL** = extensor carpi radialis longus; **EDC** = extensor digitorum communis; **FCR** = flexor carpi radialis; **FDS** = flexor digitorum superficialis; F**DP** = flexor digitorum profundus; **HYO** = Sterno Hyoideus; **STER** = Sternocleidomastoideus; **SCA** = anterior scalenus; **SPL** = splenius capitis; **UTR** = upper trapezius; **LTR** = lower trapezius; **RA** = rectus abdominis; **RF** = rectus femoris; **TA** = tibialis anterior; **ES** = erector spinae; **MF** = multifidus; **GMAX** = gluteus maximus; **BF** = biceps femoris; **GM** = medial gastrocnemius; **SOL** = soleus; **GL** = lateral gastrocnemius; **VM** = vastus medialis; **VL** = vastus lateralis; **TFL** = Tensor fascia latae; **OI** = internal obliques; OE = external obliques; **IL (L3**) = ilicostalis L3 level; **LO (T12)** = longissimus at T12 level; **lBR** = brachioradialis; **ANC** = anconeus; **mBB** = medial head biceps brachii; **lBB** = lateral head biceps brachii; **Brac** = brachialis; **lTB** = lateral head triceps brachii; **longTB** = long head triceps brachii; **mDEL** = medial deltoid; **PM** = pectroalis major, **aDEL** = anterior deltoid, **pDEL** = posterior deltoid, **LD** = latissimus dorsi; **ECU** = extensor carpi ulnaris; **FCU** = flexor carpi ulnaris

**Outcomes: Hz** = hertz; **EMG** = electromyography; NNMF = non negative matrix factorization; **PCA** = principal components analysis; **MVC** = maximal voluntary contraction

### Risk of bias

The inter-rater agreement for each criterion was between 57.1% to 100% for percentage agreement, and 0.35 to 1 for Gwet’s AC1 score (Table 3). Three studies scored an overall low risk of bias [24, 43, 60], one study scored an overall unclear risk of bias [61], and three studies scored an overall high risk of bias [25, 58, 59]. The three studies which had an overall high risk of bias had a common high risk in the number of muscles (Criterion R7 in Table 3) included for matrix decomposition [25, 58, 59]. In the studies which had an overall unclear or high risk of bias, the use of concatenation versus averaging to prepare the data input matrix was not clearly reported [25, 61].

**Table 3:** External generalizability and risk of bias of included studies.

Studies	E1	E2	E3	R1	R2	R3	R4	R5	R6	R7	R8	Summary
Diamond et al. 2017	+	+	+	*	*	*	*	*	*	***	*	***
Heales et al. 2016	+	+	+	*	*	*	*	*	*	***	=	***
Gizzi et al. 2015	+	+	+	*	*	*	*	*	*	*	*	*
Wang et al. 2015	+	+	+	*	*	*	*	*	*	*	=	=
van den Hoorn et al. 2015	+	+	+	*	*	*	*	*	*	*	*	*
Manickaraj et al. 2017	+	+	+	*	*	*	*	*	*	***	*	***
Muceli et al. 2014	+	+	+	*	*	*	*	*	*	*	*	*
% Agreement	100	100	100	100	100	71.4	85.7	71.4	100	85.7	57.1	
Gwet’s AC1	1	1	1	1	1	0.67	0.84	0.62	1	0.80	0.35	

External generalizability criteria

1. E1: Population

2. E2: Motor task

3. E3: Instrumentation

Risk of Bias criteria

1. R1: Selection bias

2. R2: Performance bias

3. R3: Attrition bias

4. R4: Reporting bias

5. R5: Detecting bias: low pass filter

6. R6: Detecting bias: high pass filter

7. R7: Detecting bias: number and choice of muscles

8. R8: Detecting bias: Concatenation vs averaging

**Abbreviations: +** = yes,— = no, * = low risk, **“=“** = unclear risk, *** = high risk

### Muscle synergies

#### Number of synergies

**Fig 2:**
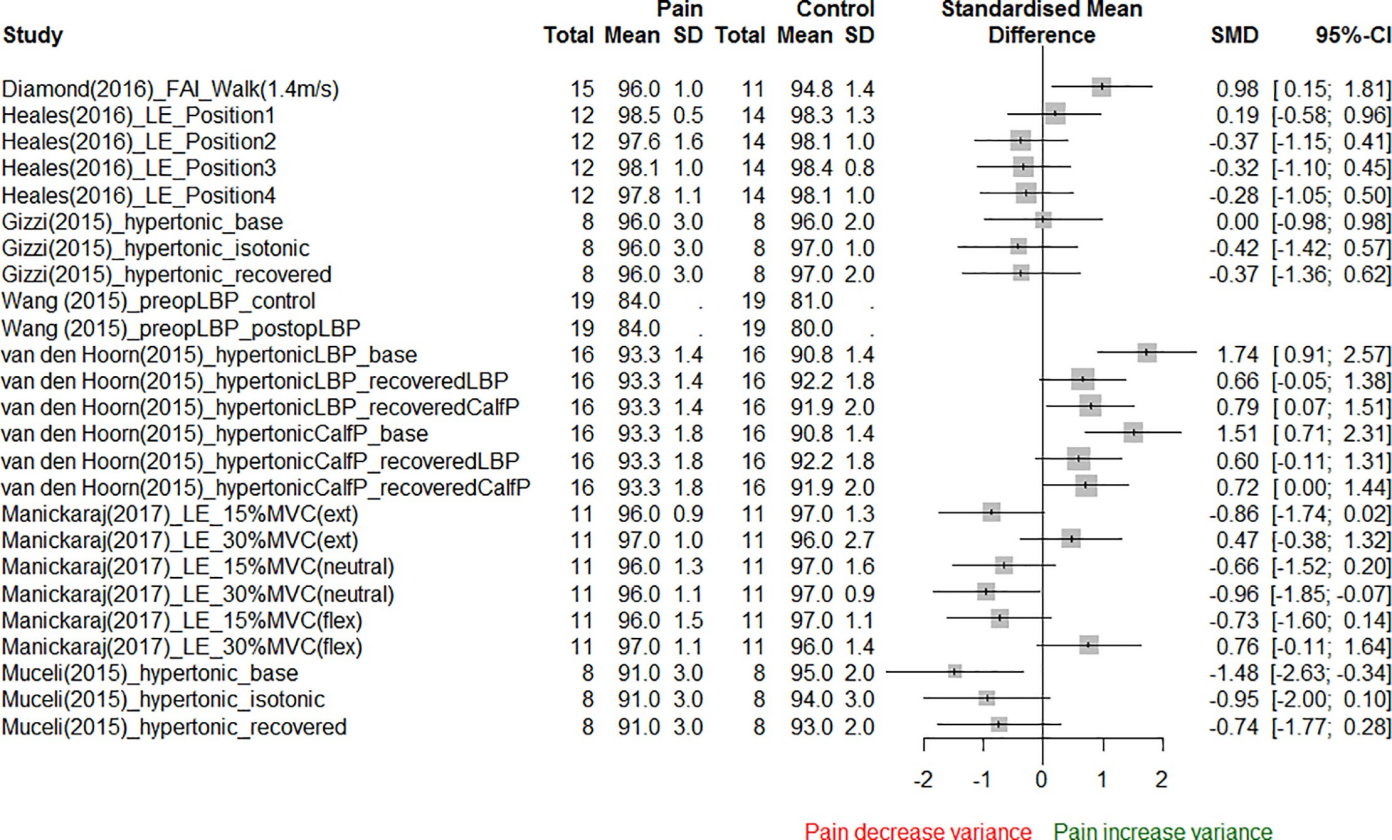
Variance accounted for by extracted muscle synergies. See S4 Table for description of study labels.

For a fixed number of extracted synergies between the pain and asymptomatic conditions, there was an unclear consistency in evidence if pain increased the VAF relative to the asymptomatic condition (Fig 2). This can be interpreted as demonstration of an unclear consistency of evidence that the number of muscle synergies needed to explain the same VAF of original muscle activation was lower with pain than in an asymptomatic condition (Fig 2, Table 4). For the same proportion of VAF to be explained, there were three studies which reported that pain reduced the number of muscle synergies [43, 59, 61], three studies which either did not report a significant difference or that significance was not reported [24, 25, 60], and one study which reported a greater number of synergies relative to the asymptomatic condition [58].

**Table 4:** Summary of findings.

Outcomes	Influence of pain			Proportion of studies (excluding NR)	Consistency
	=	NR	Δ		
Number of synergies or % VAF/ R^2^ *	(2)(3)		⤓ (1) ⤓(4) ⤓(5) ↑ (6) ↑(7)^	3/7 = 43%	Unclear
“W” weights^@^		(2)(3)(5) (7)	↑(1) ⤓(4) ^ ↑⤓(6)	1/3 = 33%	low
“C” weights^#^^&^		(2)(3)(4) (7)	↑(1) ^#^^ ↑(5)^&^ ↓(6)^&^	1/3 = 33%	low
Between conditions similarity^$^		(1)(2)(4)(6)	↓(3)^ ↓(5) ^ ↓(7) ^	3/3 = 100%	high
Reconstruction quality**		(4)(6)	↑(1) ↓(2) ↓(3)^ ↓(5) ↓(7) ^	4/5 = 80%	high

Studies: (1) = Diamond et al. (2017); (2) = Heales et al. (2016); (3) = Gizzi et al. (2015); (4) = Wang et al. (2015); (5) = van den Hoorn et al. (2015); (6) = Manickaraj et al. (2017); (7) = Muceli et al. (2014)

^ Significance not reported

*For a similar proportional variance to be explained of the original muscle activation patterns, does pain-disorder require an increase (↑) or decrease (↓) number of required synergies relative to a pain-free state?

^@^Are “W” weights of synergies with pain-disorder relative to pain-free?

^#^ Are “C” weights of synergies at similar periods, greater (↑) or lesser (↓) with pain-disorder relative to pain-free states?

^&^ Are “C” weights of synergies, delayed (↓) or earlier (↑) in pain-disorder relative to pain-free states?

^$^ Are “W” or “C” weights more (↑) or less (↓) similar in pain-disorder compared to pain-free states (Between conditions analysis)?

**When using a pain-free synergy, is the reconstructed EMG VAF in pain-disorder greater (↑) or lesser (↓) than in pain-free?

#### W and C weight differences

There was a low consistency of evidence that the W weights of muscles within the extracted synergies decreased with pain relative to an asymptomatic condition (Table 5). One study reported that muscles which weighted higher in healthy individuals, weighted lower in individuals with low back pain [61]. Another study reported that femoral acetabular impingement increased the W weighting of the obturator internus muscle in the third synergy relative to healthy controls during walking [59]. In contrast, a study on individuals with lateral epicondylalgia reported that the W weights of muscles in the first synergy was higher in those with lateral epicondylalgia compared to healthy controls, but only during a lower intensity gripping task at 15% of the maximal voluntary contraction [58].

**Table 5:** Qualitative synthesis of results.

Study	W loading	C loading	Similarity
Diamond et al. 2016	•OI in synergy 3 FAI > control (p = 0.02), in early swing of walking	•Synergy 3 (OI, QF) FAI > control early swing–p values (Not reported)	•Not reported
Heales et al. 2016	•Not reported	•Not reported	•Not reported
Gizzi et al. 2015	•Not reported	•Not reported	**“W” weights** •NDP of 4 modules between 0.69 to 0.76 in all 4 conditions (p = 0.795).Within-subject similarity greater than between-subject variability. •Control vs base (NDP = 0.91), post vs base (NDP = 0.92), control vs post (p = 0.575). Modules were similar between control and base/post. •Pain vs base (NDP = 0.79), pain vs control (p = 0.012), post vs pain (p = 0.036). Modular similarity lower in pain than base. **“C” weights** •Correlation activation coefficients homogenous across conditions (base vs control = 0.73, base vs pain 0.70, base vs post 0.69).
Wang et al. 2015	•W_PC1_ per group: Control = ES and MF loaded ≥ 0.5, LBP pre-op = TA and GM loaded ≥ 0.5, LBP post-op = TA and BF loaded ≥ 0.5 •W_PC1_ combined group: GM loaded ≥ 0.5 •	•Not applicable as time-varying EMG signals were not used	•Not reported
van den Hoorn et al. 2015	•Not reported	•Peak activation of synergy 1 occurred earlier during CalfP than control (-6.4% of the gait cycle; P < 0.01) and LBP (-4.2% of the gait cycle; P < 0.01).	•W and C weightings of synergy 1 and 5 similar between control and other 4 conditions. •W weights between control and 4 other conditions: 9%, 45%, 48%, 31%, 13% of the r-values were < 0.80 for synergies 1–5, respectively •C weights between control and 4 other conditions: 2%, 28%, 64%, 25%, 6% of the rmax-values were < 0.80 for synergies 1–5, respectively
Muceli et al. 2015	•Not reported	•Not reported	**“W” weights** •Pain condition reduced similarity of synergy 1 (average of 0.75) compared to base •Synergy 3 maintained similarity between the base and pain condition in 6/8 subjects •Synergy 2 maintained similarity between the base and pain condition in 7/8 subjects **“C” weights** •Reduced similarity between pain and base conditions for all synergies
Manickaraj et al. 2017	•15% MVC–greater W weights for all muscles in synergy 1 in LE > control (p = 0.019) •30% MVC–greater W weights for all muscles in synergy 1 in control > LE in wrist flexion (p = 0.036)	•15% MVC–time of peak C weight of synergy 1 delayed in LE compared to control in wrist extension (p = 0.028) and wrist neutral (p = 0.01) •15% MVC–time of peak C weight of synergy 2 delayed in LE compared to control in wrist extension and neutral (all P < 0.05) •30% MVC–peak C timing of synergy 2 delayed in wrist extension in LE compared to control (all P = 0.013)	•W weights similarity between synergy 1 and 2 greater in LE compared to control at 15%MVC wrist extension (P = 0.005) •C weights equally similar between synergy 1 and 2 between groups •30% MVC–W weights in synergy 1 loaded lower for all muscles in wrist flexion in LE compared to control (all P < 0.05)

Abbreviations

**Muscles: OI** = obturator internus; **QF** = quadratus femoris; **TA** = tibialis anterior; **ES** = erector spinae; **MF** = multifidus; **BF** = biceps femoris; **GM** = medial gastrocnemius

**Clinical: FAI** = femoral acetabular impingement; **LE** = lateral epicondylalgia; **LBP** = low back pain; **CalfP** = calf pain

**Assessment: EMG** = electromyography; **MVC** = maximal voluntary contraction; **W** = muscle weightings; **C** = activation coefficients; **R** = right; **NDP** = normalized dot product; **PC** = principal components

There was also a low consistency of evidence that the C weights of synergies were delayed in painful conditions relative to an asymptomatic condition (Table 5). One study reported an earlier peak activation of the first synergy during gait [43], whilst one study reported a delayed peak activation in the first and second synergies during a gripping task [58], with pain relative to an asymptomatic condition.

#### Similarity of synergies and reconstruction quality

**Fig 3:**
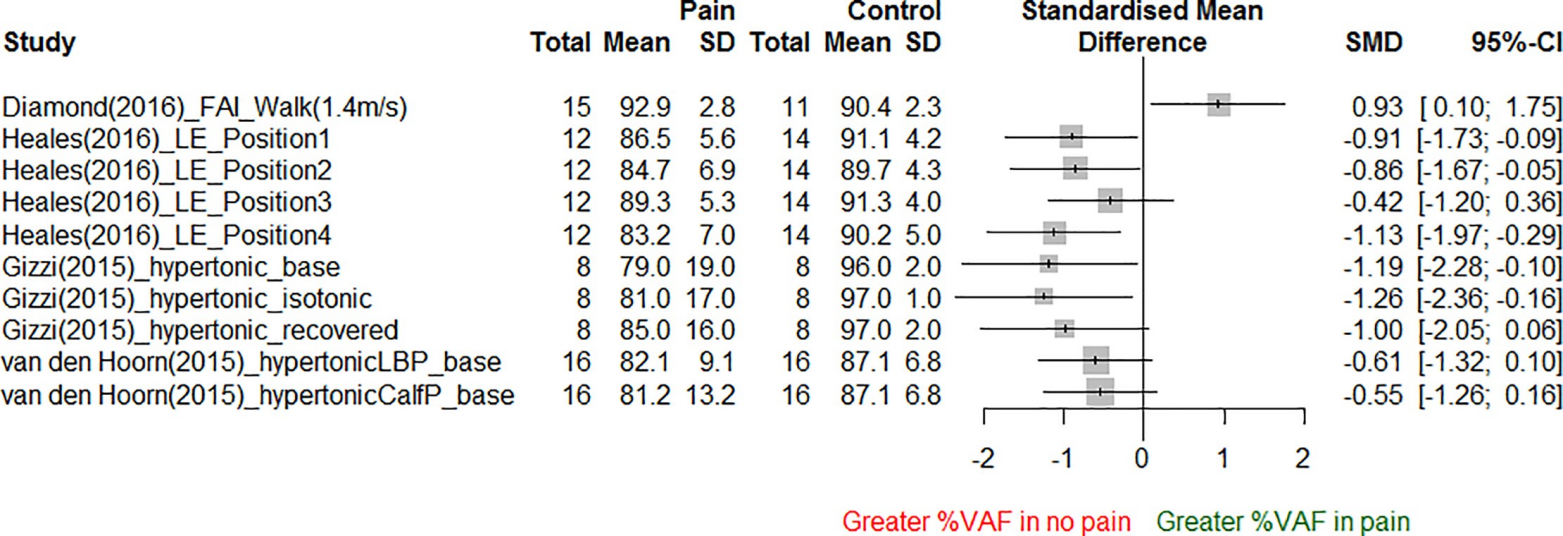
Reconstruction quality using muscle synergies from asymptotic conditions. See S4 Table for description of study labels.

Of the three studies which reported similarity measures between symptomatic and asymptomatic conditions [24, 43, 60], there was a high consistency of evidence that there was a reduced similarity between synergies when pain was present relative to an asymptomatic condition (Table 5). Four out of five studies reported a lower reconstruction quality of the original EMG signals in the pain condition compared to the asymptomatic condition, when using the muscle weightings of synergies from the asymptomatic condition [24, 25, 43, 60] (Fig 3).

## Discussion

The neural control of muscles is thought to be hierarchically organised, from individual muscles into smaller groups of muscle synergies [21]. Muscle synergy analysis using matrix decomposition is a recently introduced method for evaluating motor control in MSK pain populations. Given the hierarchical control of motor control, synergy analysis has the potential to uncover consistent patterns of altered motor control in people with MSK pain, amidst the significant variability of individual muscle activations [10, 18]. With the exception of a narrative review [8], this is the first systematic review in the area of muscle synergy analysis in MSK pain disorders.

### Influence of risk of bias on muscle synergy analysis

Two risks of bias criteria were commonly implicated in studies that scored an overall unclear or high risk of bias. These criteria were the selection of muscles and the choice of concatenation versus averaging to prepare the data input matrix [40–42]. Discussion of these two factors in the context of the present review could inform future research implementing muscle synergy analysis in MSK pain populations.

Ideally, the investigation of muscle synergies should be performed on every muscle needed in a motor task. Realistically, only a subset of muscles are investigated, as some muscles require invasive intramuscular EMG assessment–which may not always be ethically and/or practically feasible. The smaller the subset of muscles used for synergy analysis, the lesser will be the true representation of all the possible set of synergies needed in a motor task [40]. Six muscles in a hand gripping task [25, 58] may appear sufficient, but anatomically, there are 17 muscles which cross the elbow and/or wrist joint. Both Heales et al. [25] and Manickaraj et al. [58] assessed slightly different muscle groups despite assessing hand gripping in individuals with lateral epicondylalgia. This suggests that all the dominant muscle groups relevant for gripping were not included in both studies [25, 58]. Also, Diamond et al. [59] retained five out of the original eight muscles for synergy analysis. The excluded muscles were large superficial muscles, and thus inclusion may change the number of extracted muscle synergies and the associated W and C weightings [59].

Concatenating EMG signals from multiple cycles or sub-tasks, compared to averaging these signals, increases the reconstruction quality and its between-participant and within-participant reliability of the original EMG signals [41, 42]. This may be more important when reconstructing EMG signals of tasks with a large number of cycles or sub-tasks. Two studies did not explicitly report if the EMG signals across cycles and sub-tasks were concatenated or averaged [25, 61], even though EMG signals were collected from 15 gripping cycles [25] and from five forward reaching tasks [61]. It is less certain if the reduced reconstruction quality between pain status and conditions could also be influenced by the method of merging EMG signals from different cycles [25], although reconstruction quality of original EMG signals was not reported in Wang et al. [61].

### Three factors influencing the pain-synergy relationship

#### Mechanical role of muscle synergies

There was consistent evidence that pain reduced reconstruction of extracted muscle synergies, indicating that the presence of pain leads to greater variability in the extracted muscle synergies. The greater variability of synergies associated with pain is supported by consistent evidence for a reduced similarity in the muscle synergies in painful conditions compared to asymptomatic conditions (Table 4). The reduction in similarity was reported both in the W and C weightings in two studies [43, 60], but only in the W weights in one study [24]. The synergies that were influenced by experimental pain were weighted by muscles where the induction of pain was performed [43, 60]. Interestingly, synergies required for successful motor performance, such as synergy one used for propulsion and synergy five used for weight acceptance in walking [43], remained similar to the asymptomatic condition, despite pain. In contrast, pain reduced the similarity of synergies four and five which are both related to trunk mechanics [43]. The importance of trunk mechanics in walking may be more important during irregular-surfaced, than level-surfaced walking [62]. This suggests that muscle synergies with primary task related functions in a motor task are less disturbed by the presence of pain, than synergies playing secondary functions such as postural roles.

There was an unclear consistency that pain was associated with a reduction in the number of required synergies, compared to asymptomatic conditions (Table 4). A reduced number of synergies in painful conditions is similar to a common finding of inter-muscular co-activation with pain [63]. It is unlikely that the absence of pain during the motor tasks [25, 58], was the sole reason contributing to the unclear consistency in the pain-synergy number relationship. Instead, consistent relationship between pain and extracted synergy number may depend on the anatomic regional span of muscles investigated. For example, van den Hoorn et al. [43] performed synergy analysis of muscles spanning the lumbar, hip, knee, and ankle joints. Similarly, Wang et al. [61] assessed muscles spanning the lumbar, hip, and knee joints. These two studies reported a reduced number of synergies in pain versus asymptomatic conditions [43, 61]. In contrast, Gizzi et al. [24] which did not find a difference in synergy number with and without pain, restricted thoracic movements during a head-pointing task, and assessed muscles largely spanning only the cranio-cervical region. This suggests that synergy deficits during pain may better manifest across muscles spanning different anatomical regions. Anecdotally, it is common to observe kinematic compensations from the thoraco-lumbo-pelvic region during head movements in the presence of neck pain.

#### Identical motor task outcome(s)

Differences in synergistic control between symptomatic and asymptomatic conditions may consistently manifest only when motor task outcomes between conditions are similar. For example, studies which reported fewer synergies with pain compared to an asymptomatic condition, had differing walking kinematics [43], forward reach distance [61], and absolute grip force targets [58] between conditions. In contrast, studies that reported similar walking spatio-temporal patterns [59] and upper limb pointing kinematics [60] between the symptomatic and asymptomatic conditions, reported a greater extracted synergy number in the former compared to the latter. Heales et al. [25] used differing absolute grip force targets between participants with and without lateral epicondylalgia. However, the authors allowed free wrist mobility and reported that wrist extension angle during gripping was similar between groups [25]. In contrast, Manickaraj et al. [58] restricted wrist angles during force gripping. This suggests that more synergies were needed to keep the wrist extension angle consistent in those with lateral epicondylalgia, compared to the asymptomatic group. This strategy may increase the wrist extensor muscles’ lever arms to reduce muscle forces.

#### Similarity in muscle synergy weightings

The greater the similarity in the W and C weightings between synergies, the smaller the number of synergies needed to perform a task [58]. Synergies have been thought to function by exhibiting co-variation behaviour to ensure consistent motor task outcomes [64]. For example, if two synergies contribute to total grip force output, one synergy must reduce its contribution if the other synergy increases to maintain a constant force output. Manickaraj et al. [58] reported greater W similarity between the extracted synergies and a reduced number of extracted muscle synergies in the lateral epicondylalgia group performing a 15% maximal force gripping task, and not in the asymptomatic cohort or during a 30% maximal gripping task. The reduced similarity in synergies that occurred in the physically more demanding gripping task may have been a neural strategy of avoiding mechanical stress overload to the painful extensor tendon [58]. Moreover, in the more forceful task, the neural drive to the synergistic muscles may have been more distributed due an increase in the descending corticospinal inputs [65].

### Methodological considerations

The present systematic review has several strengths such as the elaboration and registration of a pre-specified protocol on PROSPERO; the use of the PRISMA checklist through the development of this systematic review and that it is the first systematic review in the area of muscle synergy analysis in MSK pain. The inherent methodological approach towards the conduct of a systematic review in laboratory-based studies on muscle synergies may however, present some limitations when drawing conclusions. First, synthesizing results from studies which investigated a heterogeneous range of pain conditions, may not appear valid, especially when sub-group analysis has often been advocated in MSK pain disorders [66]. However, the intention of this review is to infer a potential set(s) of principle by which MSK pain influences muscle coordination in movements. Synthesizing the influence of pain mechanisms on motor control across a range of MSK pain conditions has been previously performed as narrative reviews [8, 10, 67].

Second, assessing risk of bias in laboratory-based studies using frameworks originating from epidemiological studies is a challenge. It could be argued the risk of bias assessment of some studies included in the present review may have been overly strict. In defence, the authors of the present study recognise that laboratory-based studies have unique methodological features, hence a specific risk of bias checklist was designed specifically for studies using matrix decomposition methods. In addition, the approach to assess each study’s overall risk of bias was obtained from the standards in the Cochrane Handbook for Systematic Reviews of Interventions. Such an approach may be subjective, in that a study’s overall risk of bias may be categorised differently based on different assessment standards. The more valid method would be to conduct a meta-regression of each risk of bias criterion to assess its influence on the effect size of each muscle synergy outcome. This was not presently possible due to insufficient studies to enable quantitative data synthesis.

### Implications

Future studies intending to use matrix decomposition to better understand altered muscle synergies during MSK pain can benefit from two recommendations synthesized from the present review. First, synergy analysis using matrix decomposition on EMG signals may be more sensitive when assessing muscles spanning multiple anatomical regions, to detect inter-joint compensatory strategies. Second, more consistent differences between pain conditions in synergy outcomes may emerge when comparing similar motor outcomes.

The findings of the present review have three clinical implications, each related to one of the aforementioned “Three factors influencing the pain-synergy relationship” (see section prior). First, rehabilitation and assessment may need to be focused on multi-joint muscle synergies with postural roles as these maybe more affected by pain, than task directed synergies with primary mechanical roles. Muscle synergies’ postural roles may in turn help in optimizing secondary functions such as postural stability and enhancing energetic efficiency–functions which contribute to a task’s overall performance and safety. Second, clinicians may be able to alter the structure of muscle synergies by manipulating the mechanical demand (e.g. increasing walking speed) and/or external mechanical support of a task. Lastly, the structure of muscle synergies may be modulated by not only pain, but also the anticipation and avoidance of pain. This may mean that the assessment and intervention of mal-adaptive psychological factors (e.g. excessive fear avoidance) can benefit the overall rehabilitation of healthy muscle synergies. To this end, more research is needed to underpin the relationship between psychological factors, pain, and the structure of muscle synergies.

## Conclusion

Based on the findings of the present review, there is consistent evidence for differences in synergy similarity and reconstruction quality of muscle synergies between asymptomatic individuals and those with MSK pain. However, there was low or unclear consistency in evidence that the differences in muscle synergies occurred at the number of synergies extracted or the W and C weightings of the extracted muscle synergies. The magnitude and direction of the effect sizes observed for the muscle synergy changes in symptomatic conditions should be interpreted with caution, given that six out of the seven included studies had an unclear or high overall risk of bias. The hierarchical nature of human motor control makes muscle synergy analysis a useful method for understanding altered motor control in the presence of MSK pain.

## Supporting information

S1 File PRISMA checklist. (DOC)

S2 File SynRevCentral_1_081217.txt. (TXT)

S3 File SynRevCinahl_1_081217.ris. (RIS)

S4 File SynRevEmbase_1_081217.ovd. (OVD)

S5 File SynRevMedline_1_081217.ovd. (OVD)

S6 File SynRevPsychINFO_1_081217.ovd. (OVD)

S7 File SynRevPubmed_1_081217.nbib. (NBIB)

S8 File SynRevPubmed_2_081217.nbib. (NBIB)

S1 Table MeSH and keyword search terms. (DOCX)

S2 Table Generalizability checklist. (DOCX)

S3 Table Risk of bias checklist. (DOCX)

S4 Table Description of labels within Figs 2 and 3. (DOCX)
