# Supplementary material for: The influence of musculoskeletal pain disorders on muscle synergies—A systematic review
Source: PLoS One. 2018 Nov 5;13(11):e0206885. doi: 10.1371/journal.pone.0206885 (PMC6218076; doi:10.1371/journal.pone.0206885)
Supplement: S1 Table — (DOCX) [file pone.0206885.s009.docx]

**S1 Table. Medical Subject Headings (MeSH) and keywords used for each term**

| **Terms** | **MeSH (where appropriate)** | **Keywords (Title/Abstract)** |
| --- | --- | --- |
| Pain | Pain | Pain |
| EMG | Electromyography | Electromyography  Electromyographic  “Muscle activity”  “Muscle activities”  EMG |
| Synergy |  | Synergy  Modular  Modules  Module  Coordination  Variability |
| Humans | Humans |  |
| Example of PubMed search string:  (("Pain"[Mesh] OR "pain")) AND ("Electromyography"[Mesh] OR electromyography OR electromyographic OR EMG OR "muscle activity" OR "Muscle activities") AND ("synergy" OR "coordination" OR "variability" OR modules OR module OR modular) AND (Humans[Mesh]) | | |
