# Supplementary material for: The influence of musculoskeletal pain disorders on muscle synergies—A systematic review
Source: PLoS One. 2018 Nov 5;13(11):e0206885. doi: 10.1371/journal.pone.0206885 (PMC6218076; doi:10.1371/journal.pone.0206885)
Supplement: S2 Table — (DOCX) [file pone.0206885.s010.docx]

**S2 Table. Checklist for external validity**

| **Experimental conduct** | | | |
| --- | --- | --- | --- |
| **Types of bias** | **Was it reported** | **Rationale/Description** | **Yes/No** |
| Population | **Yes/No**  If Yes: copy and paste the sentence/section which indicates so. | Clinical studies  Are there sufficient details to determine if the results can be generalized to participants external to the study with a similar condition?  Experimental pain studies  Are there sufficient details to determine if pain induction protocol can be replicated |  |
| Intervention (motor task) | **Yes/No**  If Yes: copy and paste the sentence/section which indicates so. | Are there sufficient details to determine if motor task protocol can be replicated |  |
| Outcome | **Yes/No**  If Yes: copy and paste the sentence/section which indicates so. | Are there sufficient details to determine if all instrumentation protocols can be replicated, with appropriate standards (e.g. EMG SENIAMS standard) |  |
