# Supplementary material for: The influence of musculoskeletal pain disorders on muscle synergies—A systematic review
Source: PLoS One. 2018 Nov 5;13(11):e0206885. doi: 10.1371/journal.pone.0206885 (PMC6218076; doi:10.1371/journal.pone.0206885)
Supplement: S3 Table — (DOCX) [file pone.0206885.s011.docx]

**S3_Table. Risk of bias assessment (Internal validity)**

| **Experimental conduct** | | | |
| --- | --- | --- | --- |
| **Types of bias** | **Was it reported** | **Rationale/Description** | **Low/Unclear/High** |
| Selection bias | **Yes/No**  If Yes: copy and paste the sentence/section which indicates so. | If baseline differences between/within groups are different apart from pain at the beginning of each experimental condition, less is known over how pain influences muscle synergy | Low   - Can a judgement be made of the similarity in baseline characteristics of patient over all conditions (e.g. all testing completed within one day)   Unclear   - A judgement cannot be made of the similarity in baseline characteristics of patient over all conditions   High   - A judgement can be made of the dissimilarity in baseline characteristics of patient over all conditions (e.g. experimental pain reduced significantly for last condition, compared to first condition) |
| Performance bias | **Yes/No**  If Yes: copy and paste the sentence/section which indicates so. | If order was not randomized, than factors such as fatigue may be systematically introduced.  If tasks were instructed to be performed differently, than the influence of pain on synergy will be obscured. | Low   - Was the order of conditions (e.g. gait speed) randomized **AND** were the motor tasks performed similarly between pain conditions   Unclear   - No mention of condition randomization **AND** no mention if motor tasks were instructed to be performed similarly   High   - Order not randomized **AND** motor task performed differently |
| Attrition bias | **Yes/No**  If Yes: copy and paste the sentence/section which indicates so. | If drop out percentage is severe, it is unknown how muscle synergies in drop-outs, would alter in the presence of pain, differently from those who are retained | Low   - <15% who did not complete all task   Unclear   - No mention of attrition or completers   High   - > 15% attrition |
| Reporting bias | **Yes/No**  If Yes: copy and paste the sentence/section which indicates so. | If reporting is only done on significant variables and not all variables, this constitutes selective reporting. | Low   - Number of DV in results == number of DV in methods/introduction   Unclear   - No mention or unclear number of DV in methods/introduction   High   - Number of DV in results < number of DV in methods/introduction |
| **Detection bias (Signal processing)** | | | |
| Low pass filtering^5,6^ | **Yes/No**  If Yes: copy and paste the sentence/section which indicates so. | The lower the LP filtering (smaller Hz), the smaller the number of synergies will be found to fulfill a prior VAF. | Low   - Did study report, or referenced a study which cites the rationale for selected LP filter?   **OR**   - Did study performed a sensitivity analysis on LP filter vs synergy number?   Unclear   - No report, or no referencing AND no sensitivity analysis on choice of LP filter   High   - Choice of LP filter, based on task-specific literature, clearly too low. |
| High pass filtering^7^ | **Yes/No**  If Yes: copy and paste the sentence/section which indicates so. | An excessively high HP filter (e.g. 250Hz) results in merged synergy modules, i.e. the smaller the number of synergies will be found to fulfill a prior VAF. | Low   - Did study report, or referenced a study which cites the rationale for selected HP filter? **OR** - Did study performed a sensitivity analysis on HP filter vs synergies   Unclear   - No report, or no referencing **AND** no sensitivity analysis on choice of HP filter   High  Choice of HP filter, based on task-specific literature, clearly too high. |
| Number and choice of muscles^8^ | **Yes/No**  If Yes: copy and paste the sentence/section which indicates so. | The smaller the number of investigated muscles, the smaller the number of extracted synergies  Previous study showed that when < 11/30 muscles used, similarity by chance < 0.8 | Low   - Was choice of number of muscles in proportion to total muscles in limb and task appropriate? **OR** - If not, were dominant muscles within limb selected?   Unclear   - Unable to discern if choice of number of muscles is appropriate;   High   - Muscles selected were not from dominant muscle groups   **OR**   - Number of muscles too low (<= 5/30) |
| Averaging, or concatenating, number of cycles ^9,10^ | **Yes/No**  If Yes: copy and paste the sentence/section which indicates so. | Averaging results in poorer reconstruction quality compared to concatenation  Concatenation of cycles < 10 poorer reconstruction quality, > 20, better reconstruction quality | Low   - Use of concatenation with adequate number of cycles (e.g. 50% of the original signal’s cycle)   Unclear   - Use of concatenation with a low number of cycles   High   - Use of averaging method to reconstruct EMG of high number of repeated cycles |
