# Supplementary material for: The influence of musculoskeletal pain disorders on muscle synergies—A systematic review
Source: PLoS One. 2018 Nov 5;13(11):e0206885. doi: 10.1371/journal.pone.0206885 (PMC6218076; doi:10.1371/journal.pone.0206885)
Supplement: S4 Table — (DOCX) [file pone.0206885.s012.docx]

**S4 Table. Description of labels of Figure 2 in manuscript**

| **Study** | **Description** |
| --- | --- |
| Diamond(2016)_FAI_Walk(1.4m/s) | Comparing Femoral Acetabular Impingement (pain) vs control during walking at 1.4m/s |
| Heales(2016)_LE_Position1 | Comparing Lateral Epicondylalgia (pain) vs control during gripping in shoulder neutral (0°), elbow flexed (90°), forearm pronated |
| Heales(2016)_LE_Position2 | Comparing Lateral Epicondylalgia (pain) vs control during gripping in shoulder neutral (0°), elbow flexed (90°), forearm neutral |
| Heales(2016)_LE_Position3 | Comparing Lateral Epicondylalgia (pain) vs control during gripping in shoulder flexed (90°), elbow extended (0°), forearm pronated |
| Heales(2016)_LE_Position4 | Comparing Lateral Epicondylalgia (pain) vs control during gripping in shoulder flexed (90°), elbow extended (0°), forearm neutral |
| Gizzi(2015)_hypertonic_base | Comparing hypertonic (pain) saline injection vs no injection (control) |
| Gizzi(2015)_hypertonic_isotonic | Comparing hypertonic (pain) saline injection vs isotonic injection (control) |
| Gizzi(2015)_hypertonic_recovered | Comparing hypertonic (pain) saline injection vs recovered condition (control) |
| Wang(2015)_preopLBP_control | Comparing low back pain before operation (pain) vs control |
| Wang(2015)_preopLBP_postopLBP | Comparing low back pain before operation (pain) vs low back pain after operation (control) |
| Van den Hoorn(2015)_hypertonicLBP_base | Comparing hypertonic (pain) saline injection to back vs no injection (control) |
| Van den Hoorn(2015)_  hypertonicLBP_recoveredLBP | Comparing hypertonic (pain) saline injection to back vs recovered back condition (control) |
| Van den Hoorn(2015)_  hypertonicLBP_recoveredCalfP | Comparing hypertonic (pain) saline injection to back vs recovered calf condition (control) |
| Van den Hoorn(2015)_hypertonicCalfP_base | Comparing hypertonic (pain) saline injection to calf vs no injection (control) |
| Van den Hoorn(2015)_  hypertonicCalfP_recoveredLBP | Comparing hypertonic (pain) saline injection to calf vs recovered back condition (control) |
| Van den Hoorn(2015)_  hypertonicCalfP_recoveredCalfP | Comparing hypertonic (pain) saline injection to calf vs recovered calf condition (control) |
| Manickaraj(2017)_LE_15%MVC(ext) | Comparing Lateral Epicondylalgia (pain) vs control during 15% maxima voluntary contraction gripping in wrist extension |
| Manickaraj(2017)_LE_30%MVC(ext) | Comparing Lateral Epicondylalgia (pain) vs control during 30% maxima voluntary contraction gripping in wrist extension |
| Manickaraj(2017)_LE_15%MVC(neutral) | Comparing Lateral Epicondylalgia (pain) vs control during 15% maxima voluntary contraction gripping in wrist neutral |
| Manickaraj(2017)_LE_30%MVC(neutral) | Comparing Lateral Epicondylalgia (pain) vs control during 30% maxima voluntary contraction gripping in wrist neutral |
| Manickaraj(2017)_LE_15%MVC(flex) | Comparing Lateral Epicondylalgia (pain) vs control during 15% maxima voluntary contraction gripping in wrist flexion |
| Manickaraj(2017)_LE_30%MVC(flex) | Comparing Lateral Epicondylalgia (pain) vs control during 30% maxima voluntary contraction gripping in wrist flexion |
| Muceli(2015)_hypertonic_base | Comparing hypertonic (pain) saline injection vs no injection (control) |
| Muceli(2015)_hypertonic_isotonic | Comparing hypertonic (pain) saline injection vs isotonic injection (control) |
| Muceli(2015)_hypertonic_recovered | Comparing hypertonic (pain) saline injection vs recovered condition (control) |
